# Supplementary material for: Drug repurposing for aging research using model organisms
Source: Aging Cell. 2017 Jun 16;16(5):1006–15. doi: 10.1111/acel.12626 (PMC5595691; doi:10.1111/acel.12626)
Supplement: Supplementary file 7 — Data S1 Zip‐Archive of all report cards. [file ACEL-16-1006-s007.zip › RC_D16.pdf]

D16

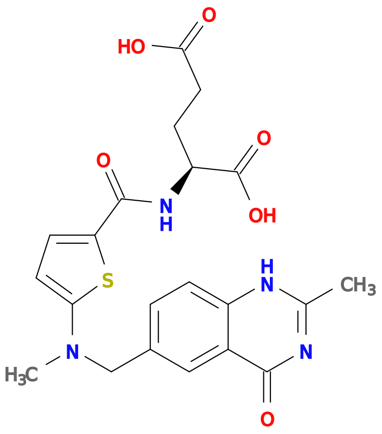

Database identifiers

ChEMBLCompound	CHEMBL225071
DrugBank	DB00293
eMolecules	2729965

Ranking

	Rank	Score
Drosophila	91/697	0.794
C. elegans	100/591	0.323

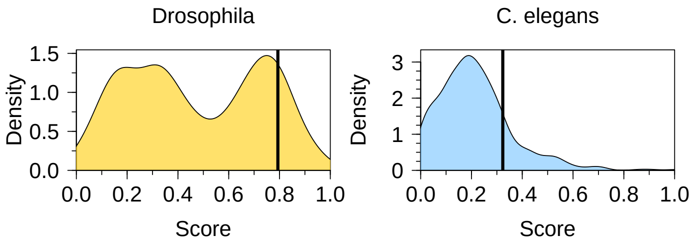

	Ageing implication	Domain conservation	Binding site conservation	Binding affinity	Bioavailability	Lipinski	Promiscuity	Purchasability	Drug approval	**Total**
Drosophila	0.792	0.944	1.0	0.933	(0.9)	0.0	-0.009	0.1	0.075	0.794
C. elegans	0.792	0.982	1.0	0.933	0.217	0.0	-0.009	0.1	0.075	0.323

Names

- Raltitrexed
- Tomudex

Roles

ChEBI entry None has no roles

Status

Approved drug (according to ChEMBL)	No
Classification (according to DrugBank)	approved, investigational
Administration Route	Intravenous
Number of Rule of 5 violations	0
Binding affinity to original target in log units (RF-Score prediction)	7.63
Burns *C. elegans* bioavailability prediction	-4.56

Medical Information

Indicatation: For the treatment of malignant neoplasm of colon and rectum
Mechanism of action: Raltitrexed is an antineoplastic Agents and folic acid antagonists. Raltitrexed inhibits thymidylate synthase (TS) leading to DNA fragmentation and cell death. It is transported into cells via a reduced folate carrier. Inside the cell Raltitrexed is extensively polyglutamated, which enhances thymidylate synthase inhibitory power and duration. Inhibition of this enzyme results in decreased synthesis of thymidine triphosphate which is required for DNA synthesis.
Toxicity: Side effects include pale skin, troubled breathing, unusual bleeding or bruising, unusual tiredness or weakness, black, tarry stools, chest pain, chills, cough, fever, painful or difficult urination, shortness of breath, sore throat, sores, ulcers, or white spots on lips or in mouth, swollen glands, increase in bowel movements, loose stools, soft stools.
Metabolism: Raltitrexed is transported into cells via a reduced folate carrier. Inside the cell it is extensively polyglutamated by the enzyme folyl polyglutamate synthetase to polyglutamate forms.
(Information from DrugBank)

Compound Target Characteristics

Thymidylate synthase

Best gene implication in ageing for this target family came from gene P45352 annotated in UniProt release 2014_02. Annotation GO 7568 (aging) was Inferred from Expression Pattern

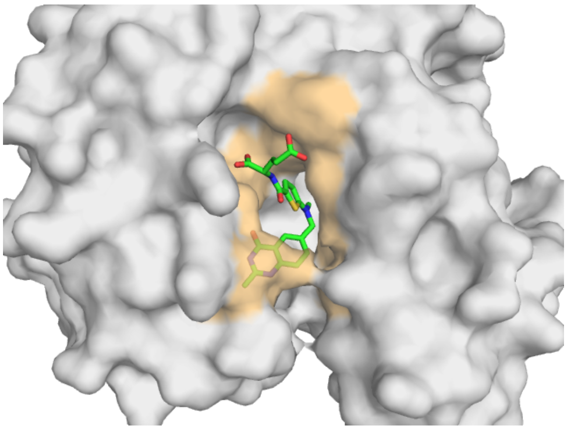

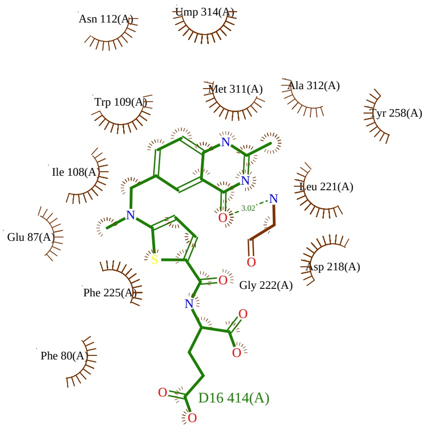

protein	amino acids contacts (binding site)
PDB:1hvy:chainA:P04818	F E I W N D L G F Y M A
PDB:1i00:chainA:P04818	F E I W N D L G F Y - -
sp:P04818:TYSY_HUMAN	F E I W N D L G F Y M A
tr:Q53Y97:Q53Y97_HUMAN	F E I W N D L G F Y M A
sp:P45352:TYSY_RAT	F E I W N D L G F Y M A
sp:P07607:TYSY_MOUSE	F E I W N D L G F Y M A
tr:Q544L2:Q544L2_MOUSE	F E I W N D L G F Y M A
tr:Q541C9:Q541C9_DROME	F E I W N D L G F Y M A
sp:O76511:TYSY_DROME	F E I W N D L G F Y M A
tr:Q9Y052:Q9Y052_CAEEL	Y E I W N D L G F Y M A
tr:Q9N588:Q9N588_CAEEL	Y E I W N D L G F Y M A
sp:P06785:TYSY_YEAST	F E I W N D L G F Y M S

	whole protein		domain-based		contact-based	
protein	ident	simil	ident	simil	ident	simil
PDB:1hvy:chainA:P04818	1.0	1.0	1.0	1.0	1.0	1.0
PDB:1i00:chainA:P04818	1.0	1.0	1.0	1.0	0.83	0.67
sp:P04818:TYSY_HUMAN	1.0	1.0	1.0	1.0	1.0	1.0
tr:Q53Y97:Q53Y97_HUMAN	1.0	1.0	1.0	1.0	1.0	1.0
sp:P45352:TYSY_RAT	0.88	0.95	0.92	0.98	1.0	1.0
sp:P07607:TYSY_MOUSE	0.88	0.95	0.93	0.98	1.0	1.0
tr:Q544L2:Q544L2_MOUSE	0.88	0.95	0.93	0.98	1.0	1.0
tr:Q541C9:Q541C9_DROME	0.58	0.8	0.67	0.88	1.0	1.0
sp:O76511:TYSY_DROME	0.58	0.8	0.67	0.88	1.0	1.0
tr:Q9Y052:Q9Y052_CAEEL	0.6	0.82	0.67	0.89	0.92	0.98
tr:Q9N588:Q9N588_CAEEL	0.6	0.81	0.67	0.89	0.92	0.98
sp:P06785:TYSY_YEAST	0.57	0.79	0.61	0.84	0.92	0.92

Ts (FBgn0024920) associated phenotypes

neuroanatomy defective

(Information from FlyBase)

Ts (UniProt:O76511) annotation

Pathway: Pyrimidine metabolism; dTTP biosynthesis.
Subunit: Homodimer.

(Information from UniProt)

WBGene00022455 associated phenotypes

lethal, sterile

(Information from WormBase)

Thymidylate synthase

Best gene implication in ageing for this target family came from gene P45352 annotated in UniProt release 2014_02. Annotation GO 7568 (aging) was Inferred from Expression Pattern

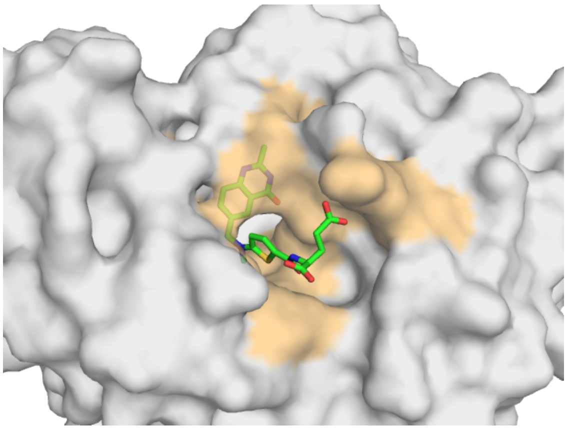

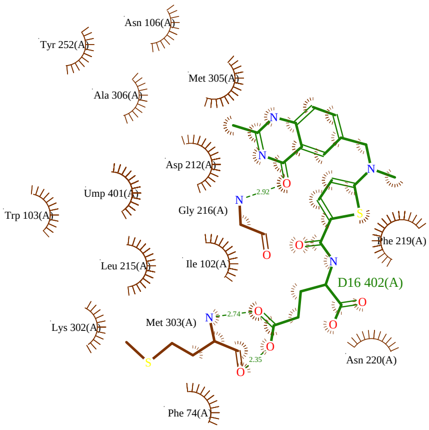

protein	amino acids contacts (binding site)														
PDB:4eb4:chainA:P07607	F	I	W	N	D	L	G	F	N	Y	I	K	M	M	A
tr:Q53Y97:Q53Y97_HUMAN	F	I	W	N	D	L	G	F	N	Y	I	K	M	M	A
sp:P04818:TYSY_HUMAN	F	I	W	N	D	L	G	F	N	Y	I	K	M	M	A
sp:P45352:TYSY_RAT	F	I	W	N	D	L	G	F	N	Y	I	K	M	M	A
sp:P07607:TYSY_MOUSE	F	I	W	N	D	L	G	F	N	Y	I	K	M	M	A
tr:Q544L2:Q544L2_MOUSE	F	I	W	N	D	L	G	F	N	Y	I	K	M	M	A
tr:Q541C9:Q541C9_DROME	F	I	W	N	D	L	G	F	N	Y	I	Q	M	M	A
sp:O76511:TYSY_DROME	F	I	W	N	D	L	G	F	N	Y	I	Q	M	M	A
tr:Q9Y052:Q9Y052_CAEEL	Y	I	W	N	D	L	G	F	N	Y	I	P	M	M	A
tr:Q9N588:Q9N588_CAEEL	Y	I	W	N	D	L	G	F	N	Y	I	P	M	M	A
sp:P06785:TYSY_YEAST	F	I	W	N	D	L	G	F	N	Y	I	Q	M	M	S

	whole protein		domain-based		contact-based	
protein	ident	simil	ident	simil	ident	simil
PDB:4eb4:chainA:P07607	1.0	1.0	1.0	1.0	1.0	1.0
tr:Q53Y97:Q53Y97_HUMAN	0.88	0.95	0.91	0.96	1.0	1.0
sp:P04818:TYSY_HUMAN	0.88	0.95	0.91	0.96	1.0	1.0
sp:P45352:TYSY_RAT	0.97	0.99	0.98	1.0	1.0	1.0
sp:P07607:TYSY_MOUSE	1.0	1.0	1.0	1.0	1.0	1.0
tr:Q544L2:Q544L2_MOUSE	1.0	1.0	1.0	1.0	1.0	1.0
tr:Q541C9:Q541C9_DROME	0.62	0.84	0.65	0.86	0.93	0.96
sp:O76511:TYSY_DROME	0.62	0.84	0.65	0.86	0.93	0.96
tr:Q9Y052:Q9Y052_CAEEL	0.6	0.81	0.63	0.85	0.87	0.92
tr:Q9N588:Q9N588_CAEEL	0.59	0.8	0.63	0.85	0.87	0.92
sp:P06785:TYSY_YEAST	0.58	0.79	0.61	0.83	0.87	0.9

Ts (FBgn0024920) associated phenotypes

neuroanatomy defective

(Information from FlyBase)

Ts (UniProt:O76511) annotation

Pathway: Pyrimidine metabolism; dTTP biosynthesis.
Subunit: Homodimer.

(Information from UniProt)

WBGene00022455 associated phenotypes

lethal, sterile

(Information from WormBase)

Thymidylate synthase

Best gene implication in ageing for this target family came from gene P45352 annotated in UniProt release 2014_02. Annotation GO 7568 (aging) was Inferred from Expression Pattern

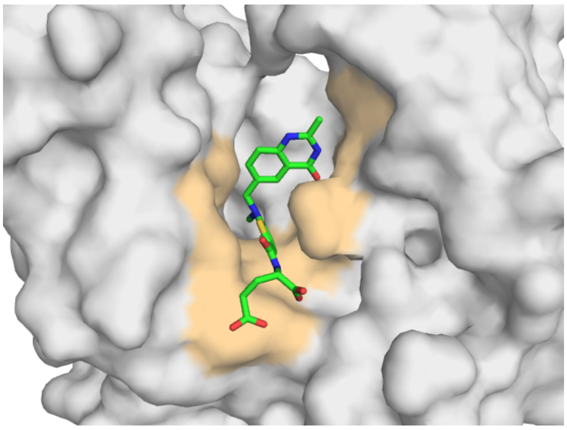

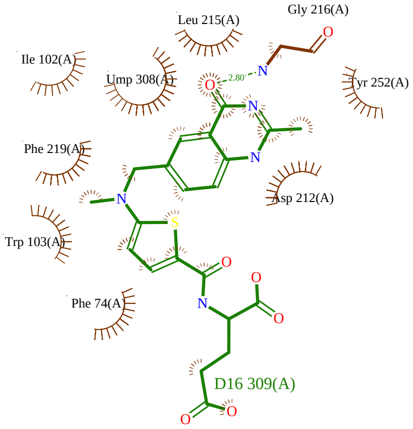

protein	amino acids contacts (binding site)
PDB:1rts:chainA:P45352	R F I W D L G F Y
PDB:2tsr:chainA:P45352	R F I W D L G F Y
tr:Q53Y97:Q53Y97_HUMAN	R F I W D L G F Y
sp:P04818:TYSY_HUMAN	R F I W D L G F Y
sp:P45352:TYSY_RAT	R F I W D L G F Y
sp:P07607:TYSY_MOUSE	R F I W D L G F Y
tr:Q544L2:Q544L2_MOUSE	R F I W D L G F Y
tr:Q541C9:Q541C9_DROME	R F I W D L G F Y
sp:O76511:TYSY_DROME	R F I W D L G F Y
tr:Q9Y052:Q9Y052_CAEEL	R Y I W D L G F Y
tr:Q9N588:Q9N588_CAEEL	R Y I W D L G F Y
sp:P06785:TYSY_YEAST	K F I W D L G F Y

	whole protein		domain-based		contact-based	
protein	ident	simil	ident	simil	ident	simil
PDB:1rts:chainA:P45352	1.0	1.0	1.0	1.0	1.0	1.0
PDB:2tsr:chainA:P45352	1.0	1.0	1.0	1.0	1.0	1.0
tr:Q53Y97:Q53Y97_HUMAN	0.88	0.95	0.9	0.96	1.0	1.0
sp:P04818:TYSY_HUMAN	0.88	0.95	0.9	0.96	1.0	1.0
sp:P45352:TYSY_RAT	1.0	1.0	1.0	1.0	1.0	1.0
sp:P07607:TYSY_MOUSE	0.97	0.99	0.98	1.0	1.0	1.0
tr:Q544L2:Q544L2_MOUSE	0.97	0.99	0.98	1.0	1.0	1.0
tr:Q541C9:Q541C9_DROME	0.62	0.84	0.65	0.85	1.0	1.0
sp:O76511:TYSY_DROME	0.62	0.84	0.65	0.85	1.0	1.0
tr:Q9Y052:Q9Y052_CAEEL	0.6	0.81	0.62	0.83	0.89	0.97
tr:Q9N588:Q9N588_CAEEL	0.59	0.8	0.62	0.83	0.89	0.97
sp:P06785:TYSY_YEAST	0.58	0.81	0.61	0.84	0.89	0.97

Ts (FBgn0024920) associated phenotypes

neuroanatomy defective

(Information from FlyBase)

Ts (UniProt:O76511) annotation

Pathway: Pyrimidine metabolism; dTTP biosynthesis.
Subunit: Homodimer.

(Information from UniProt)

WBGene00022455 associated phenotypes

lethal, sterile

(Information from WormBase)

Thymidylate synthase

Best gene implication in ageing for this target family came from gene P45352 annotated in UniProt release 2014_02. Annotation GO 7568 (aging) was Inferred from Expression Pattern

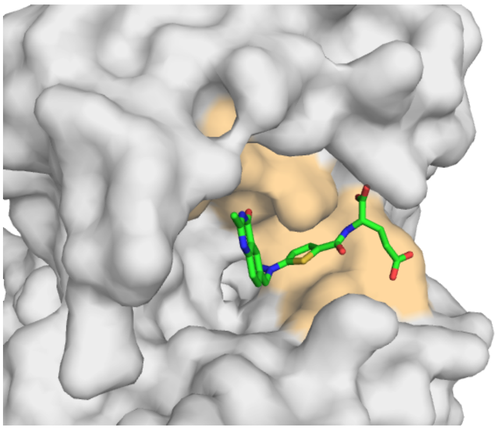

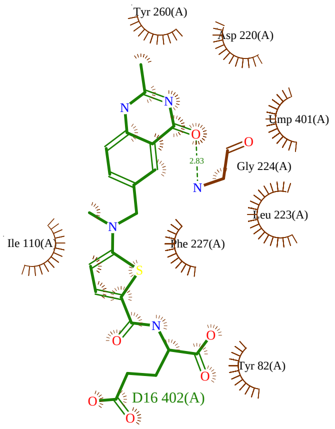

protein	amino acids contacts (binding site)						
PDB:4iqq:chainA:Q9Y052	Y	I	D	L	G	F	Y
tr:Q53Y97:Q53Y97_HUMAN	F	I	D	L	G	F	Y
sp:P04818:TYSY_HUMAN	F	I	D	L	G	F	Y
sp:P45352:TYSY_RAT	F	I	D	L	G	F	Y
sp:P07607:TYSY_MOUSE	F	I	D	L	G	F	Y
tr:Q544L2:Q544L2_MOUSE	F	I	D	L	G	F	Y
tr:Q541C9:Q541C9_DROME	F	I	D	L	G	F	Y
sp:O76511:TYSY_DROME	F	I	D	L	G	F	Y
tr:Q9Y052:Q9Y052_CAEEL	Y	I	D	L	G	F	Y
tr:Q9N588:Q9N588_CAEEL	Y	I	D	L	G	F	Y
sp:P06785:TYSY_YEAST	F	I	D	L	G	F	Y

	whole protein		domain-based		contact-based	
protein	ident	simil	ident	simil	ident	simil
PDB:4iqq:chainA:Q9Y052	1.0	1.0	1.0	1.0	1.0	1.0
tr:Q53Y97:Q53Y97_HUMAN	0.62	0.86	0.66	0.88	0.86	0.97
sp:P04818:TYSY_HUMAN	0.62	0.86	0.66	0.88	0.86	0.97
sp:P45352:TYSY_RAT	0.62	0.85	0.65	0.87	0.86	0.97
sp:P07607:TYSY_MOUSE	0.62	0.85	0.65	0.87	0.86	0.97
tr:Q544L2:Q544L2_MOUSE	0.62	0.85	0.65	0.87	0.86	0.97
tr:Q541C9:Q541C9_DROME	0.56	0.84	0.61	0.87	0.86	0.97
sp:O76511:TYSY_DROME	0.56	0.84	0.61	0.87	0.86	0.97
tr:Q9Y052:Q9Y052_CAEEL	1.0	1.0	1.0	1.0	1.0	1.0
tr:Q9N588:Q9N588_CAEEL	0.99	0.99	1.0	1.0	1.0	1.0
sp:P06785:TYSY_YEAST	0.52	0.78	0.56	0.83	0.86	0.97

Ts (FBgn0024920) associated phenotypes

neuroanatomy defective

(Information from FlyBase)

Ts (UniProt:O76511) annotation

Pathway: Pyrimidine metabolism; dTTP biosynthesis.
Subunit: Homodimer.

(Information from UniProt)

WBGene00022455 associated phenotypes

lethal, sterile

(Information from WormBase)
